# Predictors of knowledge level and awareness towards the principles and methodology evaluation of pharmacoeconomics in Saudi Arabia

**DOI:** 10.1080/20523211.2024.2442496

**Published:** 2025-01-09

**Authors:** Dhafer Mahdi Alshayban

**Affiliations:** Department of Pharmacy Practice, College of Clinical Pharmacy, Imam Abdulrahman Bin Faisal University, Dammam, Saudi Arabia

**Keywords:** Pharmacist, pharmacoeconomics, pharmaceutical expenditure

## Abstract

**Background:**

Pharmaceutical expenditure has been a major concern for decision-makers worldwide. One strategy to control medication costs involves applying pharmacoeconomic (PE) methods in the approval and listing of new medications. Pharmacists need to possess the knowledge, skill, and competence to analyse and implement PE study findings. This study aimed to evaluate the predictors of pharmacy professionals’ knowledge and awareness towards PE and their ability to apply its concepts. Furthermore, this study sought to identify the barriers to the practical application of PE.

**Methods:**

A cross-sectional study was conducted between October 2022 and June 2023. A chi-square test was employed to examine the associations between dependent and independent variables while multiple binary logistic regression was performed to identify predictors of PE knowledge and awareness.

**Results:**

A total of 204 pharmacists were included. The mean age was 29.34 years (SD: 6.45) and 75.5%of participants were male. Of the participants, 46.6% held a bachelor’s degree. A total of 81.4% were aware of PEs and 65.2% exhibited low knowledge levels. Around 60% of participants agreed to all the questions concerning the pharmacists’ attitudes towards PE. Females were 2.6 times more likely than males to have good PE knowledge (AOR = 2.62, *p* < 0.01). Participants aged 26–35 and those over 36 were 2.6 times (AOR = 2.6, *p* < 0.05) and 2.8 times (AOR = 2.83, *p* < 0.01) more likely to have good knowledge than those under 25, respectively.

**Conclusion:**

This study highlighted a gap in the knowledge, and ability to apply PE concepts in practice. Key predictors such as being aged 30 or older, having longer years of work experience, and holding advanced pharmacy degrees were linked to higher levels of PE knowledge, positive attitudes, and awareness and enhanced the ability to apply PE concept. Future research should assess the effectiveness of PE courses offered in pharmacy colleges across Saudi Arabia.

## Background

Assessing the clinical effectiveness of new healthcare interventions, including pharmaceuticals, is crucial for understanding their impact within the clinical environment. However, various considerations such as medication cost must be accounted for when introducing new medications (Alhussien et al., 2024; Bootman et al., 2005; Drummond et al., 2005). Therefore, in the decision-making process, evidence for different outcomes and costs should be assessed and compared. Pharmacoeconomics (PE) is used to identify and compare different types of costs and medication outcomes to inform policymakers about the cost-effectiveness of interventions (Alzarea et al., 2022; Bootman et al., 2005; Drummond et al., 2005; Jayasree et al., 2016). PE attempts to answer the question of whether the added benefit of one intervention is worth the added cost of that intervention. In other words, PE recognises, measures, and compares the input and output of new and existing treatments in healthcare systems and society (Farid & Baines, 2021; Jayasree et al., 2016; Raj et al., 2020; Savkar et al., 2014).

There are four methods used in conducting PE studies (Bootman et al., 2005; Drummond et al., 2005): cost–benefit, where both costs and outcomes are assigned monetary values so that costs and benefits can be easily compared; cost-effectiveness, where outcomes are measured in clinical units; cost-utility, where therapeutic outcomes are measured in quality-adjusted life years (QALY); and cost minimisation, considered partial PE evaluation because it assesses whether two medications are similar in terms of efficacy, safety, and tolerability (Bootman et al., 2005; Drummond et al., 2005; Jayasree et al., 2016; Raj et al., 2020). Although PE studies are conducted in academic fields, they are crucial in optimising healthcare outcomes and resource allocation (Al-Jazairi et al., 2011; Mori et al., 2013).

With the changing roles of pharmacy professionals in recent years from focusing solely on medication dispensing to patient treatment and promoting safe and rational medication use, pharmacists have become essential healthcare professionals who must partake in the decision-making process for medications (Adunlin et al., 2023; Mori et al., 2013; Tonin et al., 2021). Therefore, pharmacists working in hospitals, community pharmacies, and other health organisations should be aware of their roles, especially their roles in kerbing healthcare expenditure (Dalton & Byrne, 2017; Rasheed et al., 2023, Rasheeda et al., 2019). Not only should they understand PE concepts such as pharmacoeconomic principles, methods, and theories, but they should know how to apply them in real practice (Al-Jazairi et al., 2011; Farid & Baines, 2021).

As pharmacoeconomic evidence gains traction in the Kingdom of Saudi Arabia, it is essential that Saudi pharmacists develop skills to critically assess, apply, and interpret economic evaluation methods. However, this competency appears to be lacking among many pharmacists globally. Pharmacists in various regions, including Gulf and Arab countries such as Jordan, generally demonstrate limited knowledge and understanding of PE [Jayasree et al., 2016; Rajkuma, 2020]. Some studies have been performed worldwide to assess pharmacists’ knowledge, awareness, and attitudes towards PE (Alhussien et al., 2024; Jayasree et al., 2016; Rajkuma, 2020; Savkar et al., 2014). However, less is known about similar outcomes in Saudi Arabia. Hence, this study sought to assess the predictors of pharmacists’ knowledge, awareness, and attitudes towards pharmacoeconomics (PE) in Saudi Arabia, along with their capability to apply its principles and evaluation methodologies. In addition, this study also aimed to evaluate the challenges associated with implementing of PE within the health care in Saudi Arabia.

## Methods

### Study design

This eight-month cross-sectional study was conducted between October 2022 and June 2023 among pharmacy professionals working in Saudi Arabia. The study included both male and female pharmacy professionals practising in Saudi Arabia who spoke either Arabic or English. Pharmacy professionals who obtained their degrees in Saudi Arabia but practiced outside of Saudi Arabia were excluded as they did not meet the inclusion criteria.

### Ethics approval and consent to participate

The study protocol and questionnaire (including the consent form) were reviewed and approved by the Institutional Review Board of Imam Abdulrahman Bin University, Dammam, Saudi Arabia, on 19-0902022 (ID: IRB 2022-05-336). Participants were requested to provide consent online before commencing the questionnaire.

### Research tool development

Data were collected using semi-structured close-ended questionnaires. The questionnaires content was developed based on a comprehensive review of literature related to pharmacoeconomic concepts and methodology, pharmacoeconomics education, and current pharmacoeconomic applications in Saudi Arabia (Al-Hemyari et al., 2023; Alhussien et al., 2024; Jayasree et al., 2016; Savkar et al., 2014; Tahashildar et al., 2015).

The final questionnaire comprised 28 questions divided into five sections. The first section included seven demographic questions about the participants. . The second section included only one awareness question while the third section assessed pharmacists’ knowledge of PE, comprising five questions (Cronbach’s alpha = 0.65). The fourth part of the questionnaire was used to assess pharmacists’ attitudes towards PE with 7 questions rated according to a five-point Likert scale (strongly agree, agree, neutral, disagree, and strongly disagree) (Cronbach’s alpha = 0.81). The final part consisted of 3 questions and was used to assess professionals’ ability to apply PE in practice settings (Cronbach’s alpha = 0.76). The questionnaire was then piloted for face validity.

### Sampling and sample collection

A convenient sampling method was used to collect samples. Data were collected through QuestionPro, an online survey using self-administrative methods. At first, contact information from pharmacy professionals was collected. Then, a questionnaire link was distributed through email, pharmacist groups on WhatsApp and Telegram, and social media (Facebook and Twitter). All participants were requested to distribute the questionnaire link to their friends and relatives in the pharmacy profession. After two weeks, a reminder email was sent to complete the questionnaire. This procedure continued until it reached the targeted sample size of 204 samples.

We assumed that a total of 195 samples might achieve more than 90% power for this unknown size of population although the actual size of population is unknown. Calculation was done using the following statistical formula.

$$n=\frac{{\left(Z_{1-\text{β}}\right)}^{2}[\;p(1-p)]}{d^{2}}$$

where *n* = required sample size, *Z*_1-β_ = Z value at power 1-β (at power 90% this value is 1.26), *p* = preferred population proportion (0.5), *d* = margin of error (ideal value is 0.05)

### Variable management

The dependent variables in the study involved participants’ levels of awareness, knowledge, and attitudes towards pharmacoeconomics (PE), along with their capacity to apply PE concepts in workplace setting. Another dependent variable is the barriers to applying PE methods. At first, a set of 5 comprehensive questions with different types of response were used to assess knowledge. These questions are: *what is PE? What are methods used in PE? What are the differences between PE methods? How are the results presented in cost-effectiveness analysis? What is the definition of Budget impact analysis*? Then, one (1) was marked for all correct responses and zero (0) for all wrong answers. The total score was calculated out of five, with higher scores representing higher levels of knowledge. The statistical k-mean cluster was used to categorise knowledge level (good/poor). The proportion of good PE knowledge at the respondent level was coded 1 and the level of poor knowledge was coded 0. A participant was classified as having good knowledge if they correctly answered at least 4 out of the 5 questions.

Only one question (response: yes/no) was used to assess PE awareness. Attitudes towards PE and the ability to apply PE concepts were assessed using 10 questions with five response options. Responses of ‘strongly agree' and ‘agree' were combined to indicate a positive attitude and/or strong ability, while the other three responses (‘neutral', ‘disagree', and ‘strongly disagree') were grouped to represent a negative attitude and/or weak ability. Respondent demographics were coded as needed.

### Data analysis

Respondent demographics and other variables were described using descriptive statistics. Frequency and corresponding percentages were reported for categorical variables while the mean with SD was presented for continuous variables. A chi-squared test was used to measure the association between dependent and independent variables. Finally, multiple binary logistic regression was conducted to determine PE knowledge and awareness predictors for pharmacy professionals. Hosmer–Lemeshow test and Pearson chi-square were applied to check the model fitness. All statistical analyses were performed in the statistical software IBM SPSS version 23 and Excel. A two-tailed *p*-value of less than 0.05 was considered significant at the 95% confidence interval (CI) level.

## Results

This study included a total of 204 pharmacists. The mean age of the subjects was 29.34 years (SD: 6.45), ranging from 21 to 51. Almost half of them (46.6%) were in the 26–35 age group and three-fourths (75.5%) were male. In terms of education level, 46.6% of pharmacists had a bachelor’s degree, followed by a PharmD degree at 29.4%, a diploma at 11.3%, a master’s at 8.8%, and residency at 2.5%, and only 1.5% were PhD degree holders. The average working experience was 5.3 years, where 36.8% of pharmacists had worked for up to 5 years. A total of 33.3% of the participants had less than one year of work experience, whereas 29.9% were employed for more than 5 years. Of those pharmacists, 44.6% worked in Ministry of Health (MOH) hospitals followed by non-MOH hospitals, which include military hospitals, security forces hospitals, national guard hospitals, and private hospitals at 41.2% and community pharmacies at 14.2% (Table 1).

**Table 1.** Demographic characteristics of the study participants (*n* = 204).

Characteristics	Frequency (*n*)	Percentage (%)
Gender		
Male	154	75.5
Female	50	24.5
Age (Year)		
(Mean ± SD) & Range	29.34 ± 6.45	21–51
20–25 years	77	37.7
26–35 years	95	46.6
> 36 years	32	15.7
Marital status		
Single	94	46.1
Married	110	53.9
Education level		
Diploma (technician)	23	11.3
Bachelor	95	46.6
PharmD	60	29.4
Master	18	8.8
Residency	5	2.5
PhD	3	1.5
Workplace		
MOH Hospitals*	91	44.6
Non-MOH Hospitals	84	41.2
Community Pharmacy	29	14.2
Practising year		
(Mean ± SD) & Range	5.3 ± 2.91`	3 months to 22 years
< 1 year	68	33.3
1–5 years	75	36.8
> 5 years	61	29.9
Nationality		
Saudi	185	90.7
Non-Saudi	19	9.3

*MOH, Ministry of health.

Table 2 shows pharmacy professionals’ knowledge and awareness levels regarding PEs, revealing that more than 80% (81.4%) of pharmacists had heard about PEs. In addition, our findings showed that two-thirds (65.2%) of pharmacy professionals demonstrated poor knowledge levels, whereas only 34.8% of participants demonstrated good knowledge.

**Table 2.** Pharmacists awareness, knowledge, attitude and ability in applying pharmacoeconomics (PE) in Saudi Arabia (*n* = 204).

Factors	Responses (*n* & %)		
Awareness	Yes	No	
Have you heard about PE?	166 (81.4)	38 (18.6)	
Knowledge	Mean (SD)	Range	
	3.79 (2.02)	0–8	
	Good knowledge	Poor knowledge	
	71 (34.8)	133 (65.2)	
Attitude	Agree	Neutral	Disagree
PE is applicable to my current pharmacy practice	135 (66.2)	41 (20.1)	28 (13.7)
Pharmacists should have some knowledge about PE	159 (77.9)	35 (17.2)	10 (4.9)
PE concept should be applied into treatment guidelines	161 (78.9)	27 (13.2)	16 (7.8)
Pharmacists should be able to provide information on appropriate use of PE methods	158 (77.5)	30 (14.7)	16 (7.8)
PE methods should be considered when assessing all new medicines'	158 (77.5)	33 (16.2)	13 (6.4)
SFDAand other health institutions should use PE analysis when setting prices for new medicines	150 (73.5)	36 (17.6)	18 (8.8)
Community pharmacists should help a patient to choose the most cost-effective medication	124 (60.8)	50 (24.5)	30 (14.7)
Ability in applying PE			
I can apply PE methods on medications that need PE evaluation	129 (63.2)	50 (24.5)	25 (12.3)
I can search for scientific sources to extract information regarding PE methods	119 (58.3)	48 (23.5)	37 (18.1)
I can interpret results of pharmacoeconomic analyses for decision-making	126 (61.8)	49 (24.0)	29 (14.2)

SFDA, Saudi Food and drug authority.

Regarding pharmacists’ attitudes towards PE, our results showed that more than 60% of pharmacy professionals agreed on all items. The maximum agreement (78.9%) was on the item ‘PE concepts should be implemented into treatment guidelines' and the minimum agreement (58.3%) was on the item ‘pharmacoeconomics are applicable to my current pharmacy practice'. Regarding their ability to apply pharmacoeconomics, most participants agreed that they are able to apply PE concepts. Around 63% of the subjects were ‘able to apply PE methods on medications needing pharmacoeconomics comparison' and almost 58% ‘could search scientific sources to extract information on pharmacoeconomics' (Table 2).

Pharmacy professional demographics and other factors were cross-tabulated to determine significant associations with knowledge and awareness levels of PEs. A significant association (*p* < 0.01) was observed between gender and knowledge. Females scored at higher proportions for both good knowledge (52.0% vs. 29.2%) and awareness (86.0% vs. 79.9%) levels than males. Among all other demographics, age, education level, working place, and year of practice were significantly associated with both variables’ knowledge and awareness levels. Descriptive statistics show that younger pharmacists had a higher proportion (61.0%) of good knowledge than others. Similar results were observed for awareness (90.9%) as well. An increasing trend observed for both knowledge and awareness was an increased education level and years of practice Compared to community pharmacies, pharmacists who work in MOH hospitals reported significantly higher proportions of good knowledge (*p* < 0.001; 26.6% vs. 13.8%) and awareness (*p* < 0.001; 89.0% vs. 51.7%). Although the proportion of good knowledge was higher (51.2%) among non-MOH hospital pharmacists, PE awareness among governmental hospital pharmacists showed higher levels than all other professionals. Nearly all attitude questions were significantly associated with knowledge except the item ‘community pharmacists should help patients choose the most cost-effective medication for him/her'. Pharmacy professionals who agreed with the attitude items found higher knowledge levels than those who disagreed. Moreover, the results revealed a significant association between PE awareness and attitude questions.

Similarly, the variables of pharmacy professionals’ ability’ to apply PE concepts were also cross-tabulated and among the three items, two items were significantly associated. Respondents who agreed reported a higher proportion of good knowledge and awareness than those who disagreed with the statement (Table 3).

**Table 3.** Association of demographic, attitude and ability variables with pharmacy professionals’ knowledge and awareness on pharmacoeconomics (PE) in Saudi Arabia (*n* = 204).

Characteristics	Knowledge		Awareness	
	Good	Poor	Yes	No
Demographic	(*n* & %)	(*n* & %)	(*n* & %)	(*n* & %)
Gender		*p* = 0.003		*p* = 0.333
Male	45 (29.2)	109 (70.8)	123 (79.9)	31 (20.1)
Female	26 (52.0)	24 (48.0)	43 (86.0)	7 (14.0)
Age (Year)		*p* < 0.001		*p* = 0.016
20–25 years	47 (61.0)	30 (39.0)	70 (90.9)	7 (9.1)
26–35 years	12 (12.6)	83 (87.4)	70 (73.7)	25 (26.3)
36 year and more	12 (37.5)	20 (62.5)	26 (81.3)	6 (18.8)
Education level		*p* < 0.001		*p* = 0.034
Diploma (Technician)	5 (21.7)	18 (78.3)	15 (65.2)	8 (34.8)
Bachelor	20 (21.1)	75 (78.9)	74 (77.9)	21 (22.1)
PharmD	33 (55.0)	27 (45.0)	53 (88.3)	7 (11.7)
Others (Master, Residency & PhD)	13 (50.0)	13 (50.0)	24 (92.3)	2 (7.7)
Working place		*p* < 0.001		*p* < 0.001
MOH Hospital	24 (26.4)	67 (73.6)	81 (89.0)	10 (11.0)
Non-MOH Hospital	43 (51.2)	41 (48.8)	70 (83.3)	14 (16.7)
Community Pharmacy	4 (13.8)	25 (86.2)	15 (51.7)	14 (48.3)
Practising year		*p* < 0.001		*p* = 0.029
Less than 1 year	21 (30.9)	47 (69.1)	49 (72.1)	19 (27.9)
1–5 years	25 (33.3)	50 (66.7)	62 (82.7)	13 (17.3)
6 years and more	25 (41.0)	36 (59.0)	55 (90.0)	6 (9.8)
Awareness		*p* < 0.001		NA
Yes	67 (40.4)	99 (59.6)		
No	4 (10.5)	34 (89.5)		
Knowledge		NA		*p* = 0.002
Good			67 (94.4)	4 (5.6)
Poor			99 (74.4)	34 (25.6)
Attitude				
PE is applicable to my current pharmacy practice		*p* = 0.001		*p* < 0.001
Agree	58 (43.0)	77 (57.0)	121 (88.1)	16 (11.9)
Disagree	13 (18.8)	56 (81.2)	47 (68.1)	22 (31.9)
Pharmacists should have some knowledge of PE		*p* = 0.002		*p* = 0.045
Agree	64 (40.3)	95 (59.7)	134 (84.3)	25 (15.7)
Disagree	7 (15.6)	38 (84.4)	32 (71.1)	13 (28.9)
PE concept should be applied into treatment guidelines		*p* = 0.032		*p* = 0.028
Agree	62 (38.5)	99 (61.5)	136 (84.5)	25 (15.5)
Disagree	9 (20.9)	34 (79.1)	30 (69.8)	13 (30.2)
Pharmacists should be able to provide information on appropriate use of PE methods		*p* = 0.020		*p* = 0.010
Agree	61 (39.1)	95 (60.9)	133 (85.3)	23 (14.7)
Disagree	10 (20.8)	38 (79.2)	33 (68.8)	15 (31.3)
PE methods should be considered when assessing all new medicines'		*p* = 0.014		*p* = 0.019
Agree	62 (39.2)	96 (60.8)	134 (84.8)	24 (15.2)
Disagree	9 (19.6)	37 (80.4)	32 (69.6)	14 (30.4)
SFDA and other health institutions should use PE analysis when setting prices for new medicines		*p* = 0.037		*p* = 0.015
Agree	58 (38.7)	92 (61.3)	128 (85.3)	22 (14.7)
Disagree	13 (24.1)	41 (75.9)	38 (70.4)	16 (29.6)
Community pharmacists should help a patient to choose the most cost-effective medication		*p* = 0.154		*p* = 0.686
Agree	48 (38.7)	76 (61.3)	102 (82.3)	22 (17.7)
Disagree	23 (28.7)	57 (71.3)	64 (80.0)	16 (20.0)
Ability of applying PE				
I can apply PE methods on medications that need PE evaluation		*p* = 0.043		*p* = 0.199
Agree	51 (39.5)	78 (60.5)	105 (81.4)	24 (18.6)
Disagree	20 (26.7)	55 (73.3)	61 (81.3)	14 (18.7)
I can search for scientific sources to extract information regarding PE		*p* = 0.637		*p* = 0.162
Agree	43 (36.1)	76 (63.9)	93 (78.2)	26 (21.8)
Disagree	28 (32.9)	57 (67.1)	73 (85.9)	12 (14.1)
I Can interpret results of pharmacoeconomic analyses for decision-making		*p* = 0.014		*p* = 0.004
Agree	52 (41.3)	74 (58.7)	119 (86.9)	18 (13.1)
Disagree	19 (24.4)	59 (75.6)	47 (70.1)	20 (29.9)

### Predictors of pharmacy professionals’ knowledge about PE

Multiple binary logistic regression models were applied to determine significant predictors of good knowledge and awareness levels among pharmacy professionals in Saudi Arabia. The results showed that females were 2.6 times more likely to have good PE knowledge than males (AOR = 2.62, *p* < 0.01). Age was significantly associated with PE knowledge. Professionals between the ages of 26 and 35 or over 36 years were 2.6 times (AOR = 2.6, *p* < 0.05) and 2.8 times (AOR = 2.83, *p* < 0.01) more likely to have good knowledge than professionals under 25, respectively. PharmD and other professionals (master’s, residency, and PhD) were 3.4 times (AOR = 3.48, *p* < 0.01) and 3.6 times (AOR = 3.60, *p* < 0.05) more likely to have good knowledge than participants whose educational degrees were diploma/technician and bachelor, respectively. No significant difference was observed in PE knowledge between diploma/technician and bachelor’s education levels. Professionals from MOH hospitals were more likely to have good knowledge than community pharmacies (AOR = 2.41, *p* < 0.01). Years of practice was a significant predictor of good knowledge level, which continued to increase with each year of professional practice. Pharmacists who knew about PEs were four times more likely to have good knowledge than their counterparts (AOR = 4.75, *p* < 0.01). Regarding ‘attitude’ variables, pharmacists who considered PEs applicable to their current pharmacy practice were 3.2 times more likely to have good knowledge than those who did not (AOR = 3.25, *p* < 0.01). Professionals who agreed with the statement, ‘Pharmacists should have some knowledge of PE’, were 3.6 times more knowledgeable than others (AOR = 3.65, *p* < 0.01). All three items in the PE methodology were significantly associated with PE knowledge. Pharmacy professionals who agreed that PE concepts should be applied to treatment guidelines (AOR = 2.37, *p* < 0.05), that pharmacists should be able to provide information on the appropriate use of PE methods (AOR = 2.44, *p* < 0.05), and that PE methods should be considered when assessing all new medicines (AOR = 2.66, *p* < 0.05) were more knowledgeable than those who disagreed. Pharmacists who agreed with the statement, ‘The SFDA and other health institutions should use PE analysis when setting prices for new medicines’, were more likely to have good knowledge than those who disagreed (AOR = 1.98, *p* < 0.05). Pharmacy professionals who could differentiate between different PE evaluation methods were more likely to have good knowledge than those who could not (AOR = 2.14, *p* < 0.05) (Table 4).

**Table 4.** Predictors of pharmacy professionals’ knowledge about pharmacoeconomics in Saudi Arabia (*n* = 204).

Characteristics	AOR	95% CI of AOR	*p*-value
Demographic			
Gender			
Male *^r^*	–	–	–
Female	2.62	1.36–5.05	0.004
Age (Year)			
Age up to 25 year *^r^*	–	–	–
Age 26–35 year	2.61	1.12–4.11	0.027
Age 36 year and more	2.83	1.06–5.38	0.003
Education level			
Diploma (Technician) *^r^*	–	–	–
Bachelor	1.04	0.32–2.90	0.429
PharmD	3.48	1.45–7.40	0.009
Others (Master, Residency & PhD)	3.60	1.03–8.17	0.045
Working place			
Community pharmacy	–	–	–
Non-MOH Hospital	2.24	0.71–7.09	0.171
MOH Hospital	2.41	2.09–7.43	0.001
Practising year			
Less than 1 year *^r^*	–	–	–
1–5 years	2.02	1.39–4.11	<0.001
6 years and more	2.10	1.22–5.03	0.008
Awareness			
No *^r^*	–	–	–
Yes	4.75	1.95–9.64	0.002
Attitude			
Pharmacoeconomics is applicable to my current pharmacy practice			
Disagree *^r^*	–	–	–
Agree	3.25	1.62–6.45	0.001
Pharmacists should have some knowledge of Pharmacoeconomics			
Disagree *^r^*	–	–	–
Agree	3.65	1.53–8.69	0.003
Pharmacoeconomics concept should be applied into treatment guidelines			
Disagree *^r^*	–	–	–
Agree	2.37	1.06–5.27	0.035
Pharmacists should be able to provide information on appropriate use of pharmacoeconomics methods			
Disagree *^r^*	–	–	–
Agree	2.44	1.13–5.26	0.023
Pharmacoeconomics methods should be consider when assessing all new medicines’			
Disagree *^r^*	–	–	–
Agree	2.66	1.19–5.88	0.016
SFDA and other health institutions should use Pharmacoeconomic analysis when setting prices for new medicines			
Disagree *^r^*	–	–	–
Agree	1.98	1.09–4.02	0.046
Ability in applying pharmacoeconomics			
I can apply PE methods on medications that need PE evaluation			
Disagree *^r^*	–	–	–
Agree	1.79	0.97–3.35	0.064
I Can interpret results of pharmacoeconomic analyses for decision-making			
Disagree *^r^*	–	–	–
Agree	2.14	1.11–4.13	0.023

AOR, adjusted odds ratio; CI, confidence interval; *r*, reference case.

### Predictors of pharmacy professionals’ awareness of PEs

Predictors of pharmacy professionals’ awareness of PEs are presented in Table 5. According to the results, demographic factors including age, education level, working place, and years of practice were significant predictors of awareness. Comparable to pharmacists’ knowledge of PEs, awareness was significantly associated with several attitude variables. One ‘attitude’ variable was significantly associated with awareness. The odds ratio revealed that pharmacy professionals who agreed with the statements were more likely to be aware than those who disagreed. The corresponding odds ratio and level of significance observed for the attitude variables were ‘PE applies to my current pharmacy practice’ (AOR = 3.48, *p* < 0.05), ‘Pharmacists should have some knowledge of PE’ (AOR = 2.17, *p* < 0.05), ‘PE concepts should be implemented into treatment guidelines’ (AOR = 2.36, *p* < 0.05), ‘Pharmacists should be able to provide information on the appropriate use of PE methods’ (AOR = 2.63, *p* < 0.05), ‘SFDA and other health institutions should use PE analysis when setting prices for new medicines’ (AOR = 2.44, *p* < 0.05), and ‘PE analysis should be considered when assessing all new medicines’ (AOR = 2.45, *p* < 0.05). The odds ratio was similar for the ability variable ‘I Can interpret results of pharmacoeconomic analyses for decision-making’ (AOR = 2.81, *p* < 0.01).

**Table 5.** Predictors of pharmacy professionals’ awareness about PE in Saudi Arabia (*n* = 204).

Characteristics	AOR	95% CI of AOR	*p*-value
Demographic			
Age (Year)			
Age up to 25 year *^r^*	–	–	–
Age 26–35 year	2.31	1.72–7.51	0.006
Age 36 year and more	1.78	0.63–5.01	0.257
Education level			
Diploma (Technician) *^r^*	–	–	–
Bachelor	1.88	0.70–5.04	0.210
PharmD	4.03	1.26–9.48	0.019
Others (Master, Residency & PhD)	6.40	1.19–12.95	0.030
Working place			
Community pharmacy *^r^*	–	–	–
Non-MOH Hospital	1.22	1.02–2.83	*p* < 0.001
MOH Hospital	2.67	1.22–5.84	0.001
Practising year			
Less than 1 year *^r^*	–	–	–
1–5 years	1.85	1.28–4.11	0.035
6 years and more	3.55	1.31–9.62	0.013
Knowledge			
Poor *^r^*	–	–	–
Good	3.75	1.91–6.49	0.002
Attitude			
PE is applicable to my current pharmacy practice			
Disagree *^r^*	–	–	–
Agree	3.48	1.68–7.20	0.012
Pharmacists should have some knowledge of PE			
Disagree *^r^*	–	–	–
Agree	2.17	1.01–4.72	0.049
PE concept should be applied into treatment guidelines			
Disagree *^r^*	–	–	–
Agree	2.36	1.08–5.13	0.031
Pharmacists should be able to provide information on appropriate use of PE methods			
Disagree *^r^*	–	–	–
Agree	2.63	1.24–5.59	0.012
SFDA and other health institutions should use PE analysis when setting prices for new medicines			
Disagree *^r^*	–	–	–
Agree	2.44	1.14–5.24	0.022
PE methods should be considered when assessing all new medicines'			
Disagree *^r^*	–	–	–
Agree	2.45	1.17–5.13	0.017
Ability in applying pharmacoeconomics			
I Can interpret results of pharmacoeconomic analyses for decision-making			
Disagree *^r^*	–	–	–
Agree	2.81	1.37–5.78	0.005

AOR, adjusted odds ratio; CI, confidence interval; *r*, reference case.

**Figure 1.**
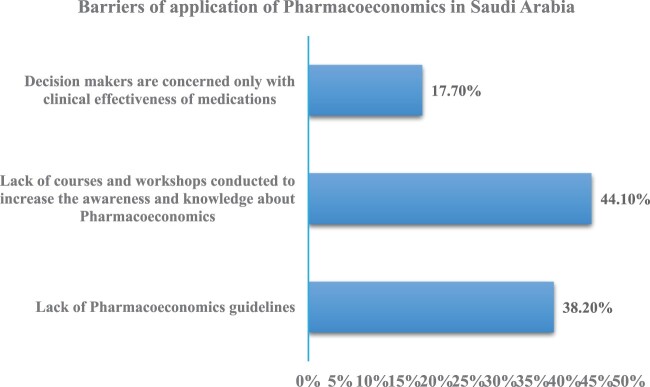
Pharmacists opinions about the barrier for application of PE in Saudi Arabia.

Figure 1 shows the participants opinions on the barriers of the PE application in Saudi Arabia. It demonstrates that the major barrier was the lack of awareness and relevant workshop conducted (44.10%). The second barrier stated as lack of PE guidelines (38.2%) and the lowest proportion observed for the point on decision-makers concerns (17.70%), as they were concentrated only on clinical effectiveness of medications instead of PE.

## Discussion

This study was conducted to investigate predictors of pharmacy professionals’ knowledge and awareness towards fundamental pharmacoeconomic propositions and evaluation methodologies in Saudi Arabia. Knowing and applying pharmacoeconomic concepts is crucial for ensuring that patients receive the optimal care at a reasonable cost, especially given the ongoing rise in pharmaceutical expenditures and budget constraints (Alhussien et al., 2024; Hammad et al., 2020; Rasheeda et al., 2019). A solid understanding of the principles and methodology of PE can play a vital role in optimising resource allocation and reducing pharmaceutical expenditure (Rasheeda et al., 2019, Rasheed et al., 2023; Tonin et al., 2021).

The results of this study highlighted two important outcomes: First, poor knowledge, a positive attitude and high awareness towards PE were observed among the participants. Second, lack of courses and training workshops related to PE were the most barriers for applying PE in real practice. Pharmacy professionals in this study had a poor knowledge overall of PE concepts. However, only a few of those surveyed had a good understanding of the principles and concepts of PE. These results were consistent with other studies results that showed poor knowledge of the basic PE concepts among pharmacists and pharmacy students (AL-Hemyari et al., 2022; Alhussien et al., 2024). Furthermore, another study suggested that most healthcare professionals, including nurses and physicians, had a poor understanding of PE methodologies and evaluation methods (Tahashildar et al., 2015). Not only poor knowledge of PE was seen in professional pharmacists but also it was observed in the postgraduate students in a tertiary care teaching hospital (Jayasree et al., 2016; Savkar et al., 2014). Poor PE knowledge may be due to several reasons: Firstly, PE is still in its infancy in Saudi Arabia. Secondly, there may be a lack of onsite scientific courses or intensive workshops offered for pharmacists in hospitals and other sectors. Thirdly, pharmacists may be unaware of the importance of PE. Nevertheless, unlike the results of the current study, one study conducted in Jordan found high knowledge among pharmacy students who participated in their study (Hammad et al., 2020).

Moreover, the present results showed significant differences in the knowledge and awareness levels between pharmacists working in the Ministry of Health (MOH) hospitals and those in community pharmacies. Community pharmacists demonstrated lower level of knowledge and awareness levels compared to pharmacists employed by the MOH hospitals. The presence of the Assistant Agency for Health Economics in MOH might be one reason for increasing the knowledge and awareness of PE amongpharmacists who work in MOH hospitals. One of the tasks of this agency is to promote research and studies related to health economics and PE. Another reason may be because community pharmacists’ focus is to provide medicine counselling and other patient-centered care services. One study reported that pharmacists in community pharmacies are usually asked by patients and other consumers to provide health-related information related to medication consultation (Farid & Baines, 2021). Unsurprisingly, participants with higher levels of education, such as PhD and residency, who might be exposed to some courses and workshops about PE, have better knowledge and awareness than those with a bachelor’s degree or diploma. However, these results conflict with another study that showed less PE knowledge among PhD students (Gupta & Malhotra, 2017; Jayasree et al., 2016; Savkar et al., 2014).

Additionally, work experience affects knowledge and awareness of PE. The findings of this study suggest that pharmacists with more years of practice had significantly better levels of knowledge and awareness than those with less than a year of practice. This finding indicates that PE concepts could be learned through experience, such as applying cost-effectiveness methods. A study by Al-Hemyari et al. found that participants’ areas of expertise had a significant statistical difference in attitudes about cost criteria used in drug formulary selection (Al-Hemyari et al., 2022).

Despite the poor PE knowledge reported among most of the participants of this study, the study findings showed that a substantial number of pharmacists had a positive attitude towards pharmacoeconomics and its applications. This result is consistent with another study, which found almost 66% of the participants had a good attitude about the criteria used in pharmacoeconomic methodology evaluations and only 2.3% had a pessimistic attitude (Tabassum et al., 2016). This optimistic perception could be due to the expected positive outcomes from applying PE to medication costs and overall healthcare expenditures. In addition, participants expressed significantly more positive attitudes towards all the items in the questionnaires, including PEs’ relevance and applicability to their clinical practice. PE concepts should be implemented in treatment guidelines and pricing medications. These findings support the emerging and expanding role of pharmacists in the clinical sector and decisions involving formularies. They also support how pharmaceuticals and other health technologies can be used more cost-effectively. In other words, the pharmacist’s role is not only dispensing and supplying medications but also collaborating with other healthcare providers in direct patient care to improve clinical outcomes and ensure the rational and cost-effective use of health resources (Tahashildar et al., 2015; Tonin et al., 2021).

The findings of this study showed that less than half of the participants indicated that they could not apply PE concepts in real life. These results are consistent with other studies, where most participants felt unprepared to apply PE principles in practice (Cati´c & Skrbo, 2013; Schmidt et al., 2023; Umair Khan et al., 2015). On the other hand, almost half of the participants surveyed could recognise and apply pharmacoeconomic methods to medications that need comparison. Searching for scientific sources to extract information about PEs allowed them to differentiate between different PE evaluation methods and I interpreting the results of pharmacoeconomic analyses for decision-making. This appreciable portion of participants believed that PE is an essential skill for Saudi pharmacists. These findings are consistent with other studies conducted in different countries (Jayasree et al., 2016; Schmidt et al., 2023; Tahashildar et al., 2015).

To support PE applications in real practice and decision-making, understanding the barriers to adopting PE concepts is vital. These barriers include a lack of nationally recognised bodies responsible for conducting pharmacoeconomic studies, a lack of national guidelines on health technology assessments (HTAs), a shortage of people with knowledge and experience in analysing and interpreting pharmacoeconomic evidence, and clinicians in hospitals disliking the concept of cost-effectiveness. The current study findings are supported by another study conducted in Saudi Arabia (Alzarea et al., 2022), which noted an absence of national governing bodies, a lack of data on medication effectiveness, an absence of pharmacoeconomic experts, and a lack of awareness about the importance of PE (Alzarea et al., 2022). Furthermore, our study results are consistent with other studies conducted in European countries where policymakers and decision-makers acted as barriers to applying PE (Cheung et al., 2018, Feig et al., 2018). One solution that suggested is to devote more hours to the PE related courses that taught at the university level (Armstrong et al., 2011; Marinova & Rascati, 2013; Soliman et al., 2013).

The current study has some limitations. Firstly, due to the cross-sectional study design, establishing a clear causal inference between the causes and the outcomes may be difficult. Generalizability is another limitation of this study. Due to convenience sampling the study sample could not be considered representative. There is a probability that the pharmacists who agreed to participate in the survey could be different from those who did not agree to participate.

## Conclusion

The results of this research highlight the key predicators of knowledge level and awareness concering the pharmacoeconomics in Saudi Arabia. Our findings indicated that participants possessed a low level of knowledge but demonstrated positive attitude and high awareness towards the principles and methodology evaluation of PE. Factors such as being aged 30 or older, female gender, longer year of practice, holding a PharmD or higher degree in pharmacy were associated with good knowledge, positive attitude and heightenedawareness. The study underscores the importance of continuous professional development in PE for pharmacists at the hospital and community levels to overcome barriers of PE application in Saudi Arabia. Future research should be directed to evaluate the topics and teaching methods of pharmacoeconomic courses at Saudi universities to ensure that graduates are prepared to apply the PE concepts in real practice.
